# Pd Clusters on Schiff Base–Imidazole-Functionalized MOFs for Highly Efficient Catalytic Suzuki Coupling Reactions

**DOI:** 10.3389/fchem.2022.845274

**Published:** 2022-03-01

**Authors:** Yangqing Liu, Jingwen Sun, Lan Fan, Qi Xu

**Affiliations:** ^1^ School of Chemistry and Chemical Engineering, Key Laboratory Under Construction for Volatile Organic Compounds Controlling of Jiangsu Province, Yancheng Institute of Technology, Yancheng, China; ^2^ Yancheng Lanfeng Environmental Engineering Technology Co., LTD, Yancheng, China

**Keywords:** Pd clusters, subnanometer size, Schiff base, Suzuki coupling reaction, metal organic frameworks

## Abstract

Subnanometer noble metal clusters have attracted much attention because of abundant low-coordinated metal atoms that perform excellent catalytic activity in various catalytic processes. However, the surface free energy of metals increases significantly with decreasing size of the metal clusters, which accelerates the aggregation of small clusters. In this work, new Schiff base–imidazole-functionalized MOFs were successfully synthesized *via* the postsynthetic modification method. Highly dispersed Pd clusters with an average size of 1.5 nm were constructed on this functional MOFs and behaved excellent catalytic activity in the Suzuki coupling of phenyboronic acid and bromobenzene (yield of biaryl >99%) under mild reaction conditions. Moreover, the catalyst can be reused six times without loss of activity. Such catalytic behavior is found to closely related to the surface functional groups that promote the formation of small Pd^0^ clusters in the metallic state.

## Introduction

Subnanometer noble metal clusters (SNMCs) with dimensions in the range from a few to dozens of angstroms have drawn focused attention (Diez and Ras, 2011; Imaoka et al., 2017; Wang et al., 2020; Yamamoto et al., 2020; Dong et al., 2021). With most of the constitutional atoms exposed to the surface, which is an unsaturated coordination environment and have higher reactivity than those in the bulk, SNMCs perform excellent catalytic activity and selectivity when serving as catalysts, thus, dramatically increasing the utilization efficiency of noble metals. The catalytic activity of SNMCs will be further improved as the size decreases, due to the surface atomic structure, electronic structure, and defects will change significantly. However, because of high surface energy, a too tiny size could lead to aggregation and particle growth (Tang et al., 2017; Si et al., 2018; Xin et al., 2018). A solution to the critical issue is to apply a nanoporous supporting material, such as graphene (Gu et al., 2017; Liu et al., 2019), carbon nanofibers (Eid et al., 2019), C_3_N_4_ (Yang et al., 2021; Zhang et al., 2021), and porous SiO_2_ (Peng et al., 2019). Nevertheless, some disadvantages, such as leaching and aggregation of SNMCs, exist during the catalytic process by using these materials as support, which, thus, weaken the catalytic activity (Gu et al., 2017; Liu et al., 2019; Zhang et al., 2021). Therefore, it is of great importance to design supporting materials that cannot only make ultrafine SNMCs with uniformity and high dispersion but also endow the obtained catalysts with excellent catalytic activity.

Metal−organic frameworks (MOFs) are a fascinating family of porous crystalline materials assembled with inorganic metal nodes and organic linkers, possessing large internal surface areas, well-defined structures, and tunable chemical properties (Lee et al., 2009; Li et al., 2012; Furukawa et al., 2013). These features confer their popular applications in heterogeneous catalysis, particularly when they are used as support for noble metal (e.g., Pd, Au, Ru, and Pt) nanoparticles (MNPs) (He et al., 2018; Han et al., 2019; Chen et al., 2021; Khan et al., 2021). On the one hand, the permanent porosity and uniform channels of MOFs make them particularly suitable for the efficient immobilization of MNPs that protect the MNPs from sintering and aggregation without inhibiting the diffusion of the reactants and products. On the other hand, the organic linkers or the metal nods of MOFs can also be used as unique functional moieties, and the interaction between MOFs and MNPs can trigger excellent synergistic effects, affording properties that are superior to the individual components (Fang et al., 2018; He et al., 2018; Mouarrawis et al., 2018; Han et al., 2019; Khan et al., 2021). Su et al. constructed a series of novel MOFs based on redox-active tetrathiafulvalene (TTF) organic linkers. Ultra-small noble metal (Ag, Pd, and Au) nanoparticles were successfully generated *in situ* and stabilized in the MOF cavity due to the reductive TTF moieties (Su et al., 2020). Recently, Guo and coworkers synthesized highly dispersed ultrafine platinum (Pt) particles with a size of <1.5 nm anchored onto amino group-functionalized MOFs (NH_2_–Ce–MOFs). It was found that the presence of –NH_2_ groups in NH_2_–Ce–MOFs played a crucial role in anchoring Pt species with high dispersion on the MOF framework (Guo et al., 2021). Based on these reports, the development of SNMC/MOF materials for noble metal catalysis will exert a significant impact on conventional chemical industrial processes.

In this work, we constructed a novel Schiff base–imidazole-functionalized MOFs and apply the MOFs as support to anchor Pd^0^. Pd^0^ clusters were uniformly dispersed in the MOFs with an average size of 1.5 nm, which is a very small size by using MOFs as support, as we know. The sub-1.5 nm Pd^0^ cluster-supported MOFs performed excellent activity in the Suzuki coupling reactions and behaved with high stability.

## Experimental Methods

### Chemicals

All chemicals and reagents were obtained from commercial sources and used as received. Specifically, zirconium chloride (ZrCl_4_), anhydrous N,N-dimethylformamide (DMF), 2-aminoterephthalic acid (H_2_BDC–NH_2_), 4-imidazolecarboxaldehyde (Im-CHO), and palladium acetate (Pd(OAc)_2_) were purchased from Aladdin.

### Catalyst Preparation

#### Synthesis of UIO-66–NH_2_

UIO-66–NH_2_ was prepared following a procedure previously reported (Zhang et al., 2016) with slight modifications. In a typical synthetic process, ZrCl_4_ (0.24 g, 1.029 mmol) was dissolved in DMF (60 ml) by sonication for 5 min. Then the ligand H_2_BDC–NH_2_ (0.186 g, 1.029 mmol) and deionized water (0.15 ml) successively dropped into the mixture, and stirring was initiated until all precursors were completely dissolved. The obtained mixture was transferred into a 100-ml Teflon-lined stainless-steel autoclave, and kept at 393 K for 24 h under static conditions. After cooled to room temperature (r.t.), the obtained yellow precipitate was separated by centrifugation at 10,000 rpm for 5 min and thoroughly washed with DMF and methanol three times, respectively. Finally, the yellow powder was freeze dried in a freeze dryer prior to experiments.

#### Synthesis of UIO-66–SB–Im

The Schiff base–imidazole-functionalized MOFs was prepared by a postmodification strategy. Typically, UIO-66–NH_2_ (0.175 g, 0.1 mmol) was dispersed in 30 ml of ethanol by sonication for 5 min. Then Im-CHO (0.144 g, 1.5 mmol) was added into the mixture, and the resulting mixture was refluxed at 80°C for 24 h. The formed yellow solid was filtered and washed with abundant ethanol three times and then freeze dried in a freeze dryer.

#### Synthesis of Pd^0^@UIO-66–SB–Im

The acetone solution of Pd(OAc)_2_ was first prepared by dissolving Pd(OAc)_2_ (0.02 g) in 5 ml of acetone. Then UIO-66-SB-Im (0.2 g) was added into the above solution, and the mixture was subsequently stirred at room temperature (r.t.) for 24 h. Afterward, the precipitate was collected by centrifugation and washed with abundant acetone three times. The as-synthesized sample was freeze dried in a freeze dryer and denoted as Pd^2+^@UIO-66–SB–Im. The Pd^2+^ in the as-synthesized sample Pd^2+^@UIO-66–SB–Im was subsequently reduced to Pd^0^ in a stream of 5% H_2_/N_2_ (75 cm^3^/min), with the temperature ramped from 20°C to 200°C (at 3°C/min) and held at 200°C for 2 h to obtain Pd^0^@UIO-66–SB–Im. For contrast, Pd^0^@UIO-66–NH_2_ was prepared through a similar process by changing the support to UIO-66–NH_2_.

### Catalyst Characterization

Pd contents were estimated on an OPTMA 20,000-V inductive coupled plasma mass spectroscopy (ICP). Elemental analyses were performed on a CHN elemental analyzer Vario EL cube. Power X-ray diffraction (PXRD) patterns were recorded on a Panalytical X’Pert^3^ Powder diffractometer with Cu Kα radiation (λ = 1.5406 Å) from 2*θ* = 5°–80°, at a scan rate of 0.2° s^−1^, with the beam voltage and current of 45 kV and 200 mA, respectively. Scanning electron microscopy (SEM) images were recorded on an FEI Nova Nano SEM 450 Prime scanning electron microscope. Transmission electron microscopy (TEM) images were obtained from a JOEL JEM-1400plus transmission electron microscope at an accelerating voltage of 200 kV. Fourier transform infrared spectroscopy (FTIR) spectra in the wavenumber range of 4,000–800 cm^−1^ were recorded on an Agilent Cary 660 FTIR Spectrometer. The nitrogen (N_2_) sorption isotherms were measured at the temperature of liquid nitrogen (77 K) by using a BELSORP-MINI analyzer with the samples being degassed at 100°C for 3 h before analysis. The surface area and pore-size distribution curves were calculated by the Brunauer–Emmett–Teller (BET) and density functional theory (DFT) method, respectively. X-ray photoelectron spectroscopy (XPS) measurement was performed on ESCALAB 250Xi with a monochromatic Mg-Kα source operated at 20 kV. All binding energies were calibrated by using the contaminant carbon (C1s = 284.8 eV) as the reference.

### Catalyst Preparation

The Pd-catalyzed Suzuki coupling reactions of phenyboronic acid and bromobenzene were carried out in a 25-ml Schlenk tube. Generally, phenyboronic acid (3 mmol), bromobenzene (2 mmol), NaHCO_3_ (4 mmol), Pd^0^@UIO-66–SB–Im (0.103 mol% Pd based on bromobenzene), dimethylacetamide (DMA, 5 ml), and 550 µl of diethyeneoxide (internal standard) were successively added into a 25-ml Schlenk tube. The tube was closed and placed in a preheated oil bath. The reaction was conducted under stirring at 90°C for 7 h. After the reaction, the tube was cooled on ice to quench the reaction, and Pd^0^@UIO-66–SB–Im was separated from the reaction medium by centrifugation and the products were analyzed by gas chromatograph [GC: Agilent GC6890N with a flame ionization detector (FID) and an HP-5 column (30 m, 0.25 mm inner diameter)]. The yield was determined with a calibration curve method.

For investigating the heterogeneous nature of the catalyst Pd^0^@UIO-66–SB–Im, a hot filtration test was applied. A mixture of phenyboronic acid (3 mmol), bromobenzene (2 mmol), NaHCO_3_ (4 mmol), dimethylacetamide (DMA, 5 ml) and the catalyst Pd^0^@UIO-66-SB-Im (0.103 mol% Pd based on bromobenzene) was stirred at 90°C. After 1.5 h, the catalyst was separated by centrifugation, the reaction solution was still stirred for 4.5 h, and the products were detected by GC at the reaction times of 0.5, 1.5, 2.5, 3.5, and 4.5 h.

In order to test the durability of the Pd^0^@UIO-66–SB–Im in the Suzuki coupling reaction system, the Pd^0^@UIO-66–SB–Im was recovered from the reaction mixture by centrifugation after the completion of previous catalytic reaction cycle, and washed with deionized water and ethanol, freeze dried in a freeze dryer, and then directly charged to the next run.

## Results and Discussion

### Structure Analysis

**SCHEME 1:**
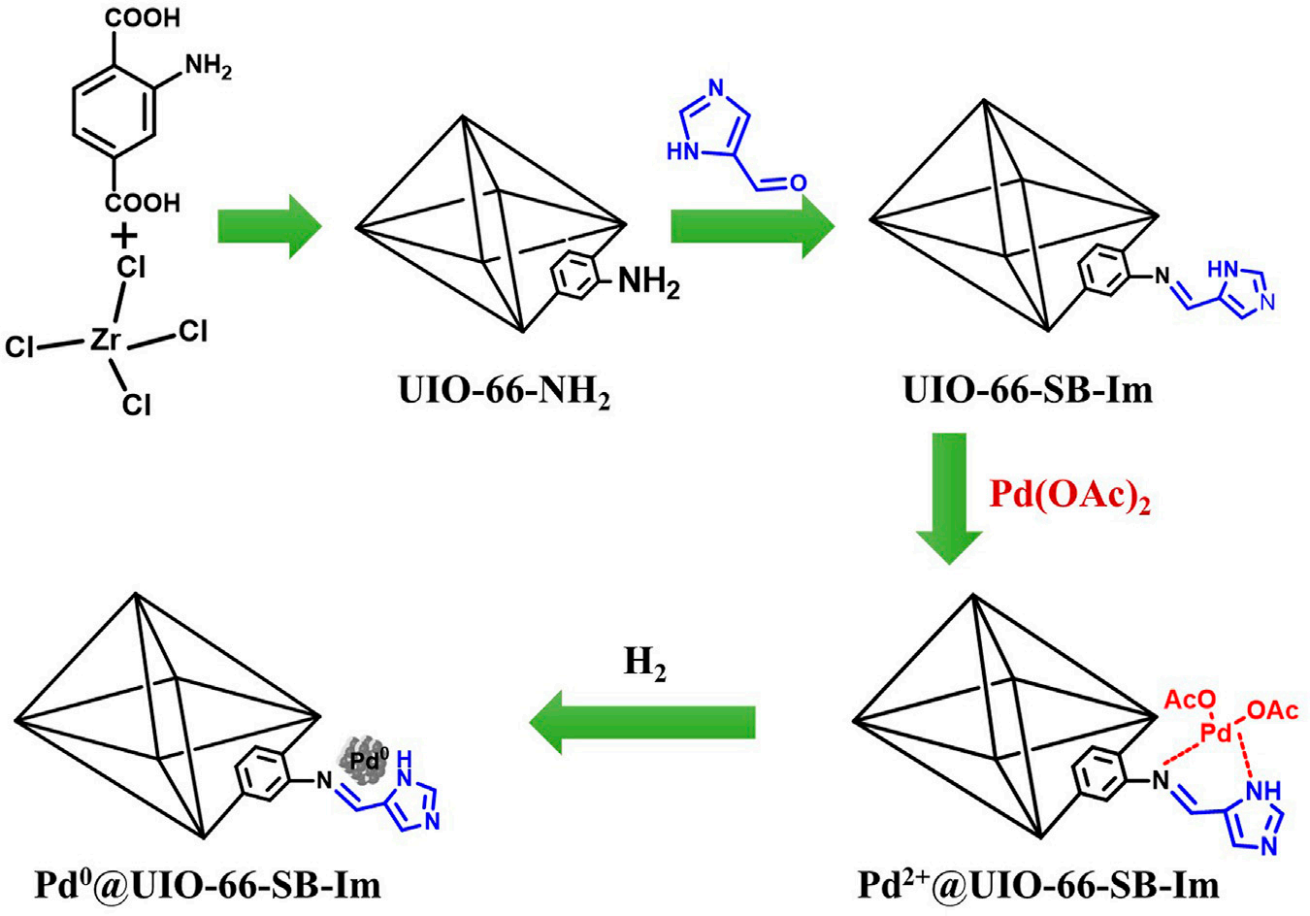
Synthetic procedure of Pd^0^@UIO-66–SB–Im.

The preparation of UIO-66–SB–Im and immobilization of Pd^0^ species to UIO-66–SB–Im are illustrated in Scheme 1. Briefly, UIO-66–NH_2_ was hydrothermally synthesized through the coordination of metal source (ZrCl_4_) and organic linker (2-aminoterephthalic acid, H_2_BDC-NH_2_), followed by an aldimine condensation process with 4-imidazolecarboxaldehyde (Im-CHO) to yield UIO-66–SB–Im support. Pd^0^@UIO-66–SB–Im catalyst was prepared by incorporating the precursor Pd(OAc)_2_ into the Schiff base–imidazole-functionalized MOFs *via* coordination and subsequent H_2_ reduction. Elemental analyses indicate that the N contents of UIO-66–NH_2_ and UIO-66–SB–Im are 4.76% and 10.23%, respectively, implying the integration of Im-CHO moieties into the UIO-66–NH_2_ matrix (Table 1). Pd^2+^@UIO-66–SB–Im and Pd^0^@UIO-66–SB–Im have a similar N content on the MOF skeleton, suggesting the stability of the surface groups and framework of UIO-66–SB–Im during the Pd immobilization process (Table 1). ICP analysis shows that Pd^0^@UIO-66–SB–Im contains a high Pd content of 2.3 wt%.

**TABLE 1:** Textural properties.

Samples	*N*%^a^	S_BET_ (m^2^/g)^b^	V_p_ (cm^3^/g)^c^	D_p_ (nm)^d^
UIO-66–NH_2_	4.76	709	0.40	2.27
UIO-66–SB–Im	10.23	537	0.30	2.24
Pd^2+^@UIO-66–SB–Im	9.58	428	0.25	2.23
Pd^0^@UIO-66–SB–Im	9.74	452	0.26	2.23

Note. a The content of *N* element.

b BET surface area.

c Total pore volume.

d Average pore size.

**FIGURE 1:**
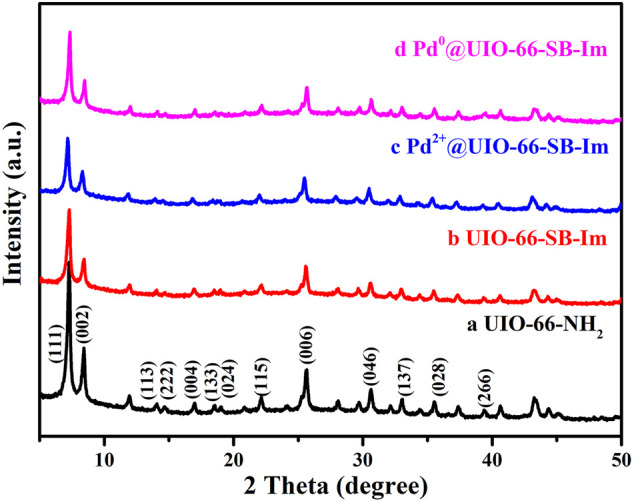
Power X-ray diffraction (PXRD) diffraction patters of UIO-66–NH_2_, UIO-66–SB–Im, Pd^2+^@UIO-66–SB–Im, and Pd^0^@UIO-66–SB–Im.

Power X-ray diffraction (PXRD) was used to characterize the crystallinity of the prepared samples. As shown in Figure 1, the experimental PXRD pattern of the synthesized UiO-66–NH_2_ matches well with the simulated one (Gang et al., 2018), indicating the successful preparation of UiO-66–NH_2_. After the aldimine condensation, the resulting product UIO-66–SB–Im performs the same PXRD pattern with UiO-66–NH_2_, suggesting that the crystallinity and structure of MIL-101 are well retained after the postsynthesis. The crystallinity and structure are still retained after loading Pd(OAc)_2_ and subsequent H_2_ reduction. Moreover, the diffraction peak of Pd nanoparticles (NPs) at 2θ = 40.1° was not detected in Pd^0^@UIO-66–SB–Im, revealing that Pd NPs could be small.

**FIGURE 2:**
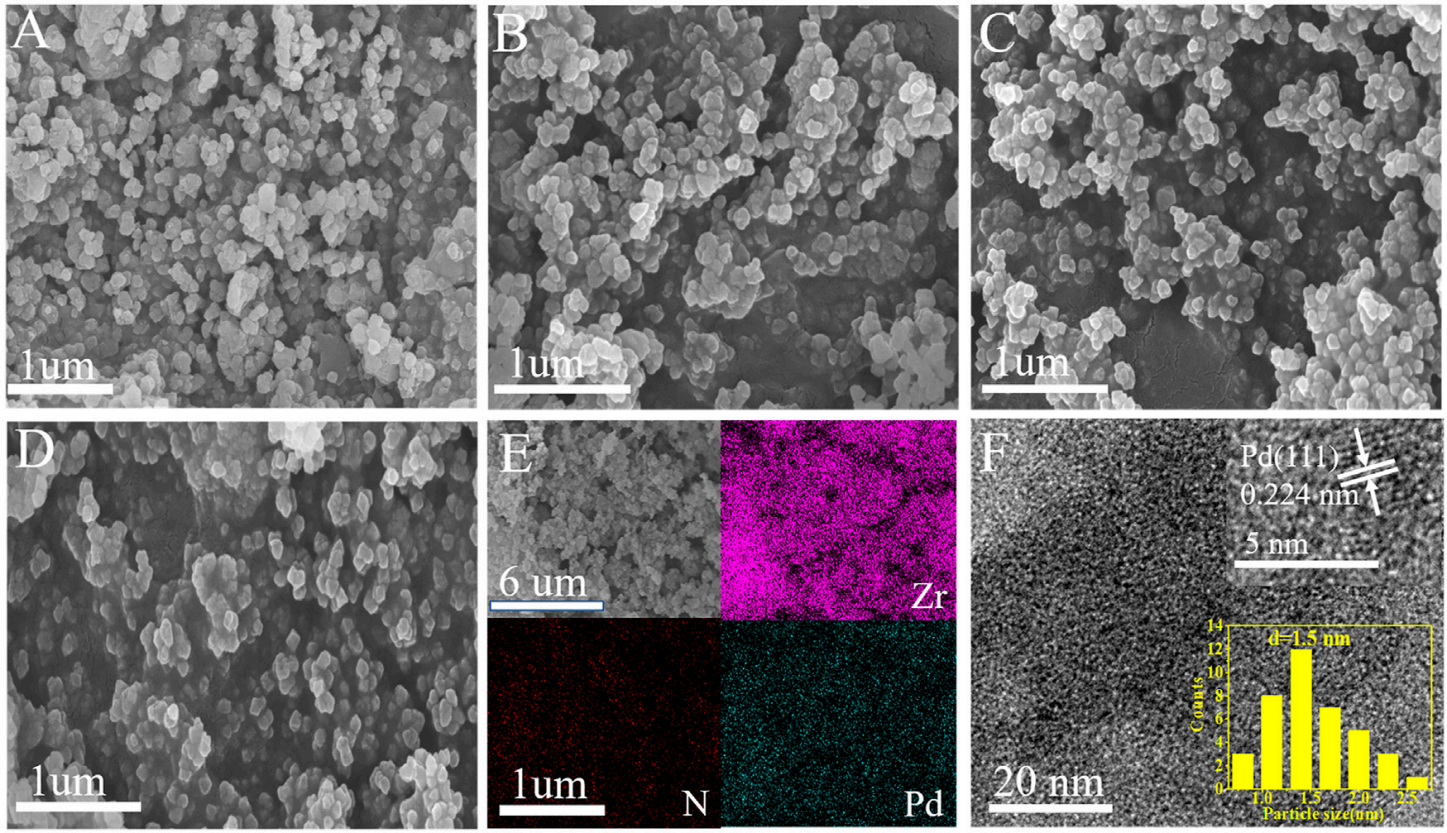
Scanning electron microscopy (SEM) images of **(A)** UIO-66–NH_2_, **(B)** UIO-66–SB–Im, **(C)** Pd^2+^@UIO-66–SB–Im, **(D)** Pd^0^@UIO-66–SB–Im, **(E)** EDS mapping images for elements Zr, N, Pd elements of Pd^2+^@UIO-66–SB–Im, transmission electron microscopy (TEM) images of **(F)** Pd^0^@UIO-66–SB–Im.

**FIGURE 3:**
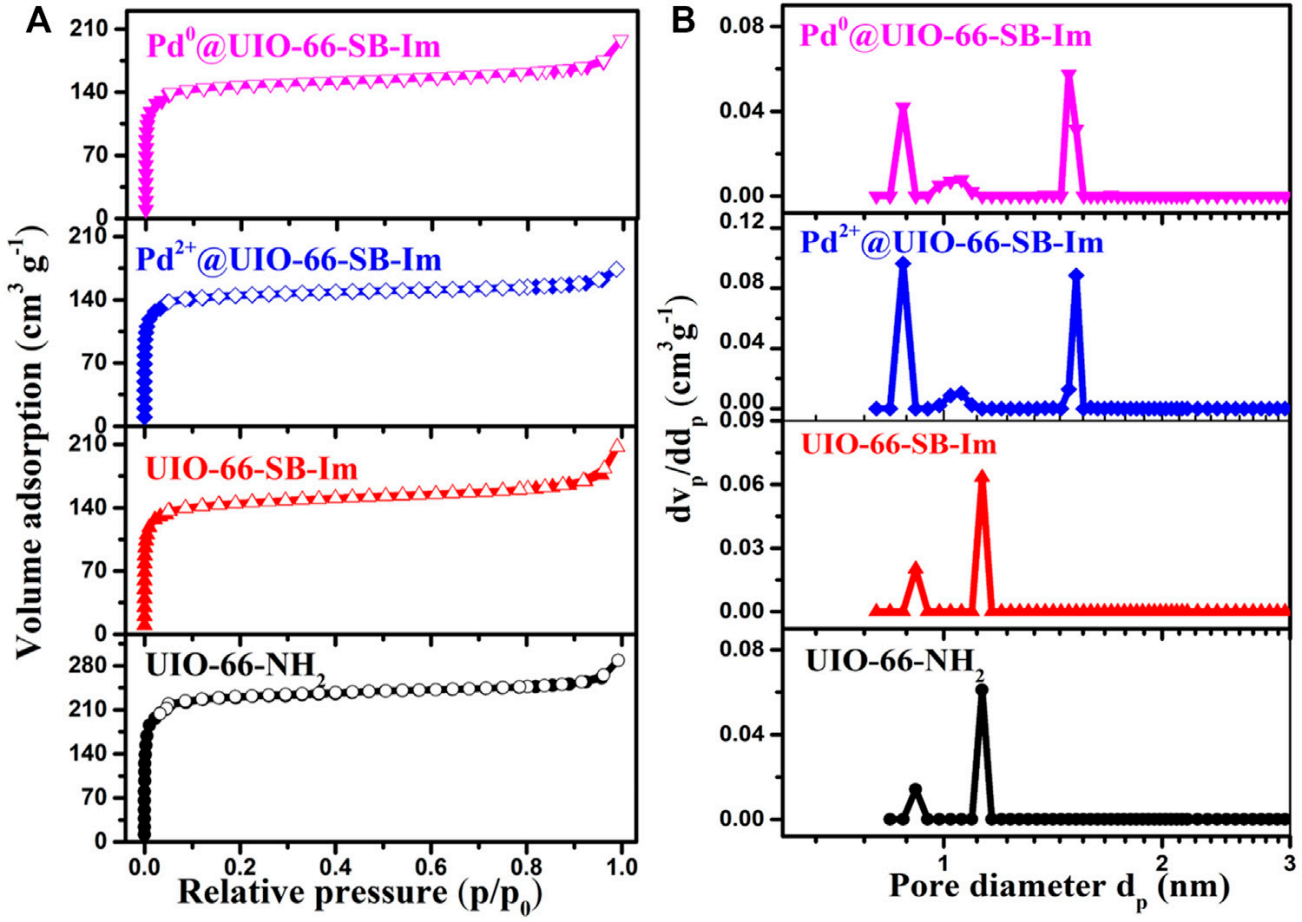
**(A)** Nitrogen (N_2_) adsorption–desorption isotherms and **(B)** pore size distribution curves of different samples.

Scanning electron microscopy (SEM) imaging shows that UIO-66–SB–Im (Figure 2A) performs similar morphology with UIO-66–NH_2_, which is composed of nanometer-leveled particles with the size in range of 100–150 nm. After loading Pd species, the morphology of Pd^2+^@UIO-66–SB–Im and Pd^0^@UIO-66–SB–Im were retained (Figures 2B, C), implying that UIO-66–NH_2_ is stable after functionalization. Elemental (Zr, N, and Pd) mapping images of Pd^2+^@UIO-66–SB–Im further prove the successful introduction of Pd species (Figure 2D). The TEM images and Pd size distribution of Pd^0^@UIO-66–SB–Im are given in Figure 3E. From the picture, the Pd clusters were uniformly distributed with average size of 1.5 nm, belonging to sub-2-nanometer size. Apart from this, the lattice spacing of Pd clusters was about 0.22 nm, ascribed to the Pd(111) crystal face (Zhao et al., 2014), which is active for Suzuki coupling reactions.

Quantitative porous properties of UIO-66–NH_2_, UIO-66–SB–Im, Pd^2+^@UIO-66–SB–Im, and Pd^0^@UIO-66–SB–Im are established by the N_2_ sorption analysis. Figure 3 illustrates the N_2_ sorption isotherms (Figure 3A) and corresponding pore size distribution curves (Figure 3B). The UIO-66–SB–Im exhibits similar N_2_ sorption isotherms with UIO-66–NH_2_ than a typical I type isotherm with a dramatic increase in nitrogen uptake in the partial pressure range of *p*/*p*_*0*_<0.1, characteristic of classical microporosity (Chen et al., 2018). The BET (Brunauer–Emmett–Teller) surface area and the total pore volume of UIO-66–SB–Im are 537 m^2^ g^−1^ and 0.3 cm^3^ g^−1^, respectively (Table 1). The decrease in the surface area and pore volume of UIO-66–SB–Im, in comparison with those of UIO-66–NH_2_ (709 m^2^ g^−1^ with a volume of 0.40 cm^3^ g^−1^), may be ascribed to the partial occupation of the cavities in NH_2_–UIO-66 by the Schiff base and imidazole groups. The pore-size distribution curve calculated by the DFT (discrete Fourier transform) method further reveals the existence of micropores with a narrow pore size distribution in UIO-66–SB–Im. After loading Pd, Pd^2+^@UIO-66–SB–Im and Pd^0^@UIO-66–SB–Im performs similar sorption isotherms with their parent UIO-66–SB–Im, revealing the preservation of porosity during the Pd-loading step. The BET surface areas of Pd^2+^@UIO-66–SB–Im and Pd^0^@UIO-66–SB–Im is 428 and 452 m^2^g^−1^, respectively, which are smaller than that of UIO-66–SB–Im due to the partial pore filling by the deposited Pd species and the weakly decreased proportion of porous structure after Pd loading (Wang et al., 2016; Liu et al., 2021). The BET surface area and pore volume of Pd^0^@UIO-66–SB–Im increased slightly with subsequent H_2_ reduction, which could be ascribed to the removal of OAc^−^ from the precursor during the reduction process (Liu et al., 2021).

**FIGURE 4:**
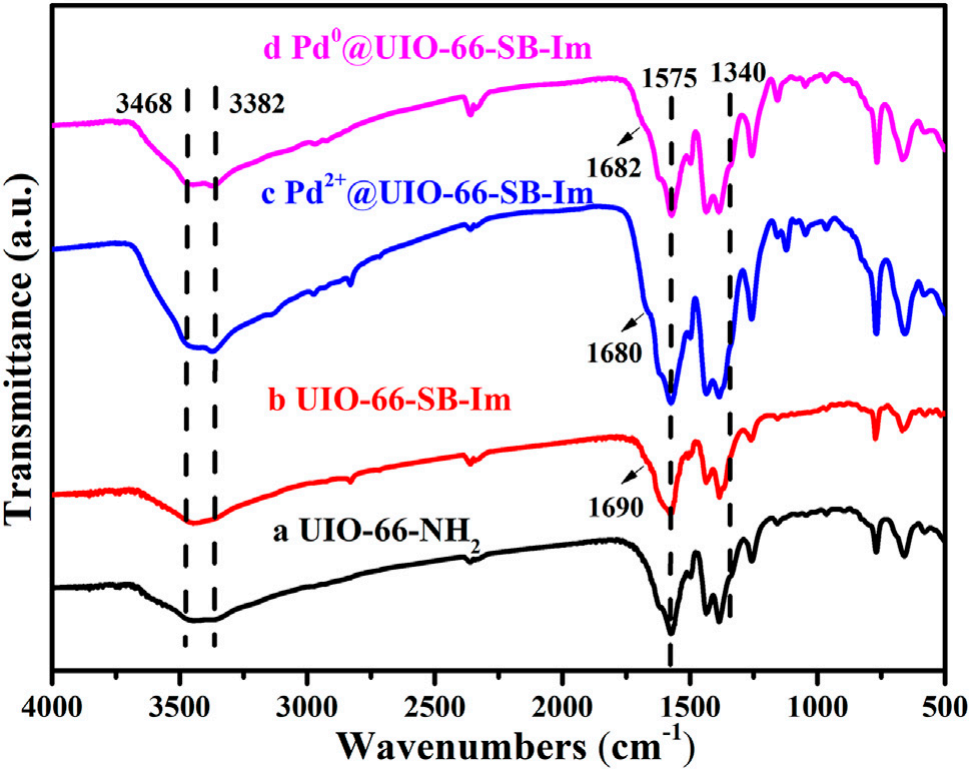
Fourier transform infrared spectroscopy (FTIR) spectra of UIO-66–NH_2_, UIO-66–SB–Im, Pd^2+^@UIO-66–SB–Im, and Pd^0^@UIO-66–SB–Im.

The structure of the samples and the relationship between Pd and the support UIO-66–SB–Im were further analyzed by Fourier transform infrared spectroscopy (FTIR). As displayed in Figure 4, the FTIR spectrum of UIO-66–NH_2_ shows a peak at 1,340 cm^−1^ that attributed to the C−N stretch vibration on benzene ring (Lin et al., 2012), while those at 3,478 and 3,371 cm^−1^ could be assigned to the asymmetric and symmetric vibrations of −NH_2_, which further demonstrate the successful synthesis of UIO-66–NH_2_ (Lin et al., 2012). After the aldimine condensation with Im-CHO, several peaks appeared in the FTIR spectrum of UIO-66-SB-Im compared with that of UIO-66–NH_2_. The new peak at 1,648 cm^−1^ could be ascribed to the stretch vibration of C=N and featured band of imidazole (Lin et al., 2012; Gang et al., 2018), indicating the successful introduction of Im-CHO and successful formation of Schiff base. After anchoring Pd(OAc)_2_ on UIO-66–SB–Im, the peak at 1,648 cm^−1^ corresponding to the Schiff base and imidazole groups is shifted to 1,675 cm^−1^, suggesting the interaction of the Schiff base and imidazole groups in UIO-66–SB–Im with Pd(OAc)_2_ (Wang et al., 2015; Liu et al., 2017). A similar phenomenon occurred after H_2_ reduction, implying the interaction of Schiff base and imidazole groups in UIO-66–SB–Im with Pd clusters.

**FIGURE 5:**
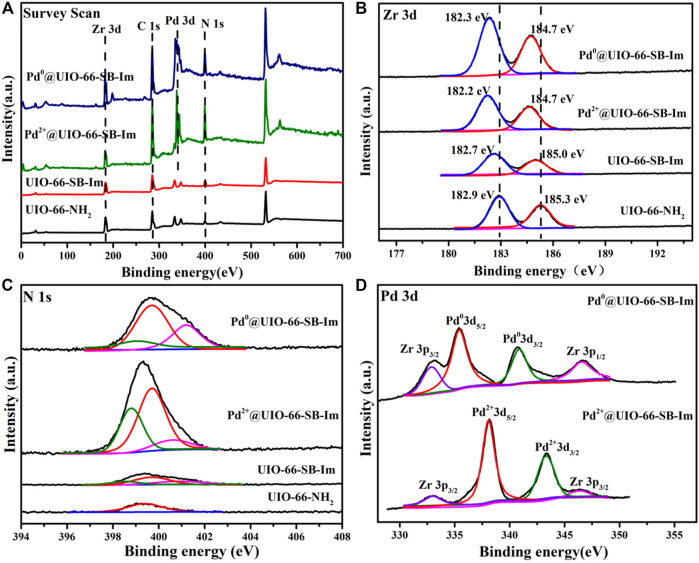
**(A)** Survey scan, **(B)** Zr 3d, **(C)** N 1s, and **(D)** Pd 3d X-ray photoelectron spectroscopy (XPS) spectra of UIO-66–NH_2_, UIO-66–SB–Im, Pd^2+^@UIO-66–SB–Im, and Pd^0^@UIO-66–SB–Im.

Surface analyses of UIO-66–NH_2_, UIO-66–SB–Im, Pd^2+^@UIO-66–SB–Im, and Pd^0^@UIO-66–SB–Im were carried out using X-ray photoelectron spectroscopy (XPS), and the results are displayed in Figure 5. Three signals were observed at the binding energies of 181.5 eV (Zr3d), 228.4 eV (C1s), and 399.1 eV (N1s) in the survey scan XPS spectrum of UIO-66–SB–Im, manifesting the existence of Zr, C, and N elements (Figure 5A) (Gang et al., 2018). The high-resolution Zr3d XPS spectrum was deconvoluted into four peaks at 182.9 and 185.3 eV, which are ascribed to Zr3d_5/2_ and Zr3d_3/2_, respectively (Figure 5B) (Gang et al., 2018). The high-resolution N1s XPS spectrum was fitted with three peaks at 398.2, 399.5, 400.6 eV, which correspond to the imine groups (−N=CH−) and imidazole N (Wang et al., 2015; Gang et al., 2018), respectively. After loading Pd, a new signal at binding energy of 338.7 eV (Pd3d) appeared in the survey scan XPS spectra of Pd^2+^@UIO-66–SB–Im and Pd^0^@UIO-66–SB–Im, further proving the successful introduction of Pd species into the support (Figure 5A). The high-resolution Zr3d XPS spectra of Pd^2+^@UIO-66–SB–Im and Pd^0^@UIO-66–SB–Im fitted likewise the two peaks with no other oxidation state of Zr peaks, indicating that the Zr was chemically stable during the functionalization of the linker and anchoring of Pd precursor (Gang et al., 2018). Moreover, compared with UIO-66–SB–Im, the peaks of Zr in Pd^2+^@UIO-66–SB–Im and Pd^0^@UIO-66–SB–Im was slightly shifted to lower energy. The phenomenon implies that the oxidation state of Zr^4+^ in Pd^2+^@UIO-66–SB–Im and Pd^0^@UIO-66–SB–Im was reduced, which may be due to the electron transfer from Pd to Zr on the support (Gang et al., 2018). The high-resolution NIs XPS spectra of Pd^2+^@UIO-66–SB–Im was fitted with three peaks at 398.5, 399.8, and 400.9 eV, which shift positively by 0.3 eV compared with UIO-66–SB–Im. The phenomenon indicates the interaction of Schiff base and imidazole with Pd (Liu et al., 2017). The high-resolution Pd3d XPS spectrum of Pd^2+^@UIO-66–SB–Im exhibits two peaks at 338.1 and 343.5 eV, assignable to Pd^2+^3d_5/2_ and Pd^2+^3d_3/2_, respectively, indicating that the surface Pd species of Pd^2+^@UIO-66–SB–Im are in a Pd^2+^ state (Figure 5D) (Liu et al., 2017; Liu et al., 2018). After reduction, Pd^0^@UIO-66–SB–Im exhibits only two Pd^0^3d peaks at 335.5 (3d_5/2_) and 340.8 eV (3d_3/2_), implying that Pd species of Pd^0^@UIO-66–SB–Im are all in the metallic state (Figure 5D) (Liu et al., 2021).

### Enhanced Catalytic Activity in the Suzuki Coupling Reaction

The catalytic performance of Pd^0^@UIO-66–SB–Im was assessed in the Suzuki coupling reaction between bromobenzene and phenylboronic acid at mild conditions. The activities of different catalysts were parallel investigated under the same reaction conditions, and the results are summarized in Table 2. The reaction does not proceed in the absence of catalysts or catalyzed by the pure support UIO-66–NH_2_ or UIO-66–SB–Im (Table 2, entries 1–3). The material Pd^0^@UIO-66–SB–Im behaved with 95.3% yield of biphenyl, indicating that the Pd^0^ species are the active species in the reaction (Table 2, entry 4). Moreover, the contrast catalyst Pd^0^@UIO-66–NH_2_ was synthesized and investigated in the reaction. Pd^0^@UIO-66–NH_2_ only performed 89.4% yield of biphenyl (Table 2, entry 5). The results show that the Pd^0^@UIO-66–NH_2_ is the best catalyst for the reaction.

**TABLE 2:** Catalytic performances of different catalysts for the Suzuki coupling reaction.

Entry^a^	Catalyst	Yield (%)
1	None	0
2	UIO-66–NH_2_	0
3	UIO-66–SB–Im	0
4	Pd^0^@UIO-66–SB–Im	95.3
5	Pd^0^@UIO-66–NH_2_	89.4

Note. a Reaction conditions: catalyst (0.103 mol% based on bromobenzene), bromobenzene (2 mmol), phenylboronic acid (3 mmol), NaHCO_3_ (4 mmol), DMA (5 ml), 7 h, 90°C.

**FIGURE 6:**
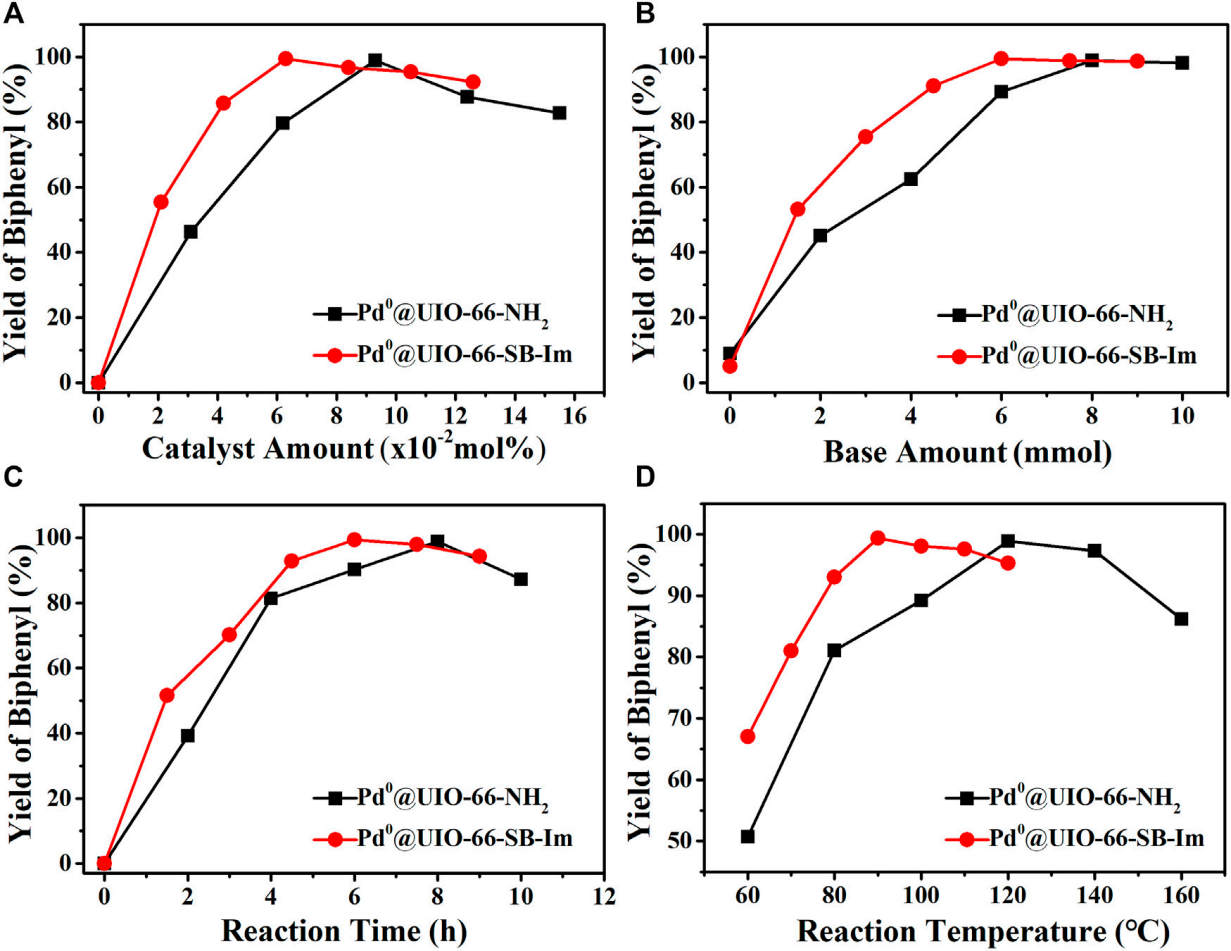
The relationship between the activity and **(A)** catalyst amount, **(B)** base amount, **(C)** reaction time, and **(D)** reaction temperature in the Suzuki coupling reaction. Pd^0^@UIO-66–SB–Im catalyzed reaction conditions: Pd^0^@UIO-66–SB-Im (0.06 mol% based on bromobenzene), bromobenzene (2 mmol), phenylboronic acid (3 mmol), NaHCO_3_ (6 mmol), DMA (5 ml), 6 h, 90°C. For each plot, a specific parameter is changed. Pd^0^@UIO-66–NH_2_ catalyzed reaction conditions: Pd^0^@UIO-66–NH_2_ (0.09 mol% based on bromobenzene), bromobenzene (2 mmol), phenylboronic acid (3 mmol), NaHCO_3_ (8 mmol), DMA (5 ml), 8 h, 120°C. For each plot, a specific parameter is changed.

The Suzuki coupling of bromobenzene and phenylboronic acid to biphenyl was further investigated by using the catalyst Pd^0^@UIO-66–SB–Im under different reactions with respect to the amount of catalyst, the amount of base, the reaction time, and the reaction temperature (Figure 6). Moreover, Pd^0^@UIO-66–NH_2_ was also parallel investigated for comparison (Figure 6). The yield as the function of the catalyst amount exhibits a “volcanic” type curve. Initially, the yield initially increases gradually when the catalyst amount increases (Figure 6A). At up to 0.063 mol%, the yield reaches the highest value of 99.4%. A further increase in the catalyst amount causes a slight decrease in yield that could be attributed to the over-oxidation of the product along with the reaction (Lin et al., 2012). For Pd^0^@UIO-66–NH_2_, more catalyst dosage (0.093 mol%) was needed to reach the highest biphenyl yield, and the yield is only 98.9%. Fixing the catalyst amount of Pd^0^@UIO-66–SB–Im at 0.063 mol%, the base NaHCO_3_ amount was studied **(** Figure 6B). A higher number of base favors higher biphenyl yield. In view of environmental impact, 6 mmol is suitable. For Pd^0^@UIO-66–NH_2_, more amount of base is needed (>8 mmol) to reach the yield of 99%, which will cause more waste of chemicals and is environmentally unfriendly (Figure 6B). The survey of the reaction time and temperature suggests that moderate reaction time (6 h) and temperature (90°C) benefit high yield of biphenyl because of the over-oxidation with elongating time or higher temperature (Figures 5E, F). The above investigation shows that Pd^0^@UIO-66–SB–Im is highly efficient for the Suzuki coupling reaction under mild condition. Compared with Pd^0^@UIO-66–NH_2_, the superior activity of Pd^0^@UIO-66–NH_2_–MC is probably ascribed to the cooperation between Pd^0^ clusters and the Schiff base–imidazole-functionalized UIO-66–SB–Im.

The scope of Pd^0^@UIO-66–SB–Im is further extended to the Suzuki coupling of various aryl halides with phenylboronic acid (Table 3). Pd^0^@UIO-66–SB–Im-catalyzed coupling of bromobenzene with electron-withdrawing groups (nitro, aldehyde, or methoxy group) performed high yields of the corresponding biphenyl products (Table 3, entries 1–3), while coupling of bromobenzene bearing electron-donating groups (methyl group) behaved with lower yields to the coupling products (Table 3, entries 4–6), implying that the withdrawing groups in bromobenzene is beneficial to the reaction, while the electron-donating groups in bromobenzene is harmful (Shang et al., 2014). Coupling of aryl iodobenzene with phenylboronic acid produced >99% yield of biphenyl (Table 3, entry 7), while coupling of chlorobenzene only obtained 37% yield of biphenyl (Table 3, entry 8) because the C–X (X = Cl, Br, I) bond dissociation energy is C–Cl > C–Br > C–I (Lin et al., 2012; Shang et al., 2014).

**TABLE 3:** Suzuki coupling reactions of various aryl halide with phenylboronic acid using Pd^0^@UIO-66–SB–Im as the catalyst.
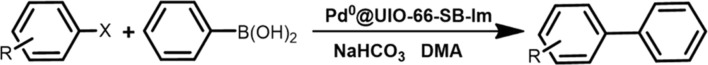

Entry^a^	Substrate	Product	Yield (%)^b^
1	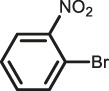	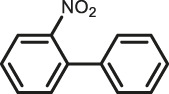	>99
2	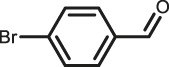	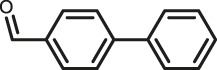	>99
3	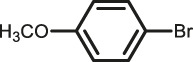	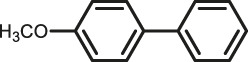	>99
4	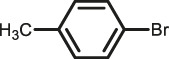	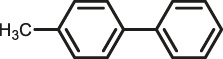	87.9
5	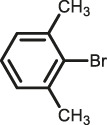	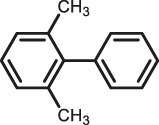	85.4
6	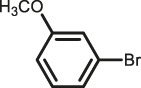	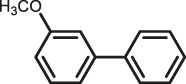	86.3
7	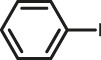	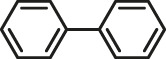	>99
8	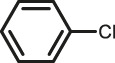	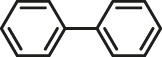	37

Note. a Reaction condition: Pd^0^@UIO-66–SB–Im (0.06 mol% based on bromobenzene), aryl halides (2 mmol), phenylboronic acid (3 mmol), NaHCO_3_ (6 mmol), DMA (5 ml), 6 h, 90°C.

b The yield of biphenyl was determined by gas chromatograph (GC) with 1,4-dioxane used as an internal standard.

**FIGURE 7:**
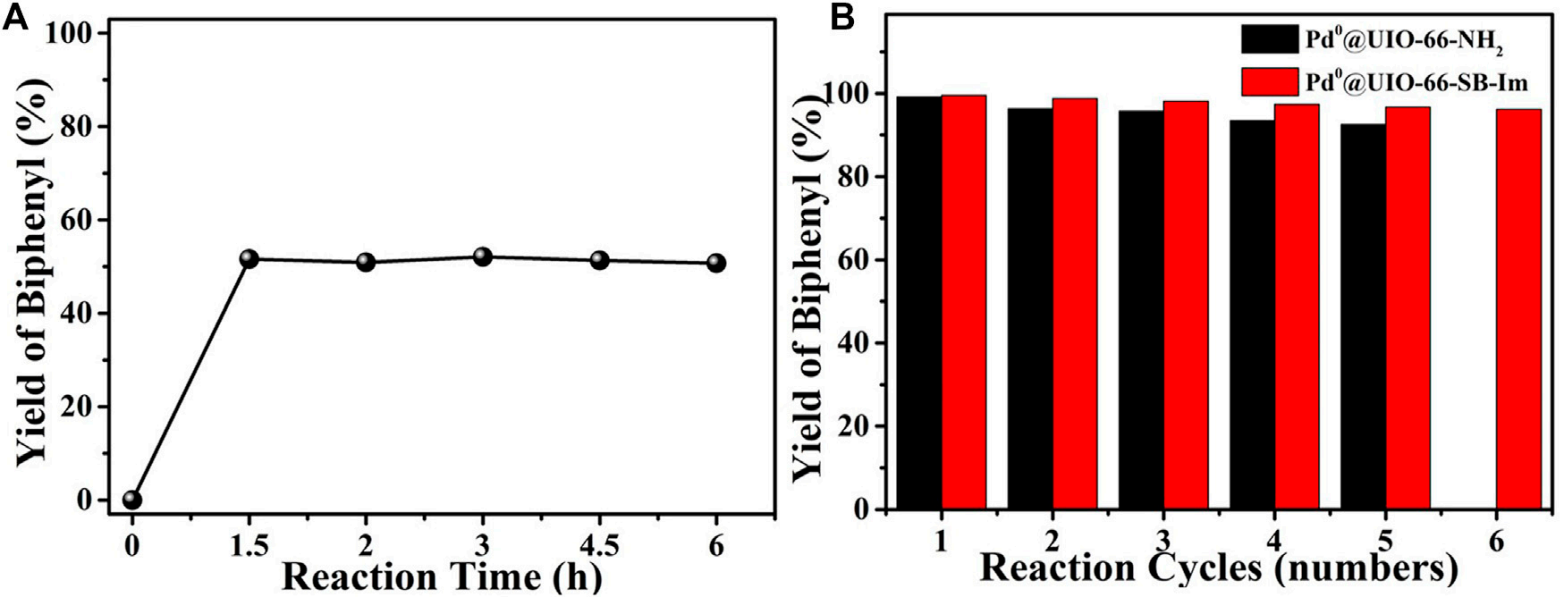
**(A)** Hot filtration test of Pd^0^@UIO-66–SB–Im catalyzed Suzuki coupling reaction. **(B)** Reusability of Pd catalysts in the catalytic Suzuki coupling reaction. (Pd^0^@UIO-66–SB–Im-catalyzed reaction conditions: Pd^0^@UIO-66–SB–Im (0.06 mol% based on bromobenzene), bromobenzene (2 mmol), phenylboronic acid (3 mmol), NaHCO_3_ (6 mmol), DMA (5 ml), 6 h, 90°C. Pd^0^@UIO-66–NH_2_ catalyzed reaction conditions: Pd^0^@UIO-66–NH_2_ (0.09 mol% based on bromobenzene), bromobenzene (2 mmol), phenylboronic acid (3 mmol), NaHCO_3_ (8 mmol), DMA (5 ml), 8 h, 120°C).

In order to investigate the heterogeneous nature of Pd^0^@UIO-66–SB–Im, a hot filtration test was carried out and is shown in Figure 7A. A reaction catalyzed by Pd^0^@UIO-66–SB–Im was prematurely stopped at 1.5 h (i.e., 51.6% biphenyl yield by GC analysis), and then the solid catalyst was removed by hot filtration. The filtrate was engaged in the additional reaction for an additional 4.5 h, and no more biphenyl was produced as analyzed by GC, confirming that the reaction proceeds on the solid surface of the catalyst and excludes the contribution of the possible leached Pd species. By simple filtration, the catalyst can be facilely separated and reused in the next time. As shown in Figure 7B, the catalyst Pd^0^@UIO-66–SB–Im could be reused for six times without significant decrease in performance, while the recycled catalyst Pd^0^@UIO-66–NH_2_ performed obvious decrease, further implying the well stability of Pd^0^ species anchored on the Schiff base–imidazole-functionalized UIO-66–SB–Im. As demonstrated by the XRD and FTIR spectra of the spent catalyst Pd^0^@UIO-66–SB–Im after six runs, the crystalline structure and the polymeric network were preserved (Supplementary Figures S1 and S2). TEM images show that the Pd^0^ clusters of Pd^0^@UIO-66-SB-Im^6th^ have grown, which may lead to the slight decrease in catalytic performance (Supplementary Figure S3).

**SCHEME 2:**
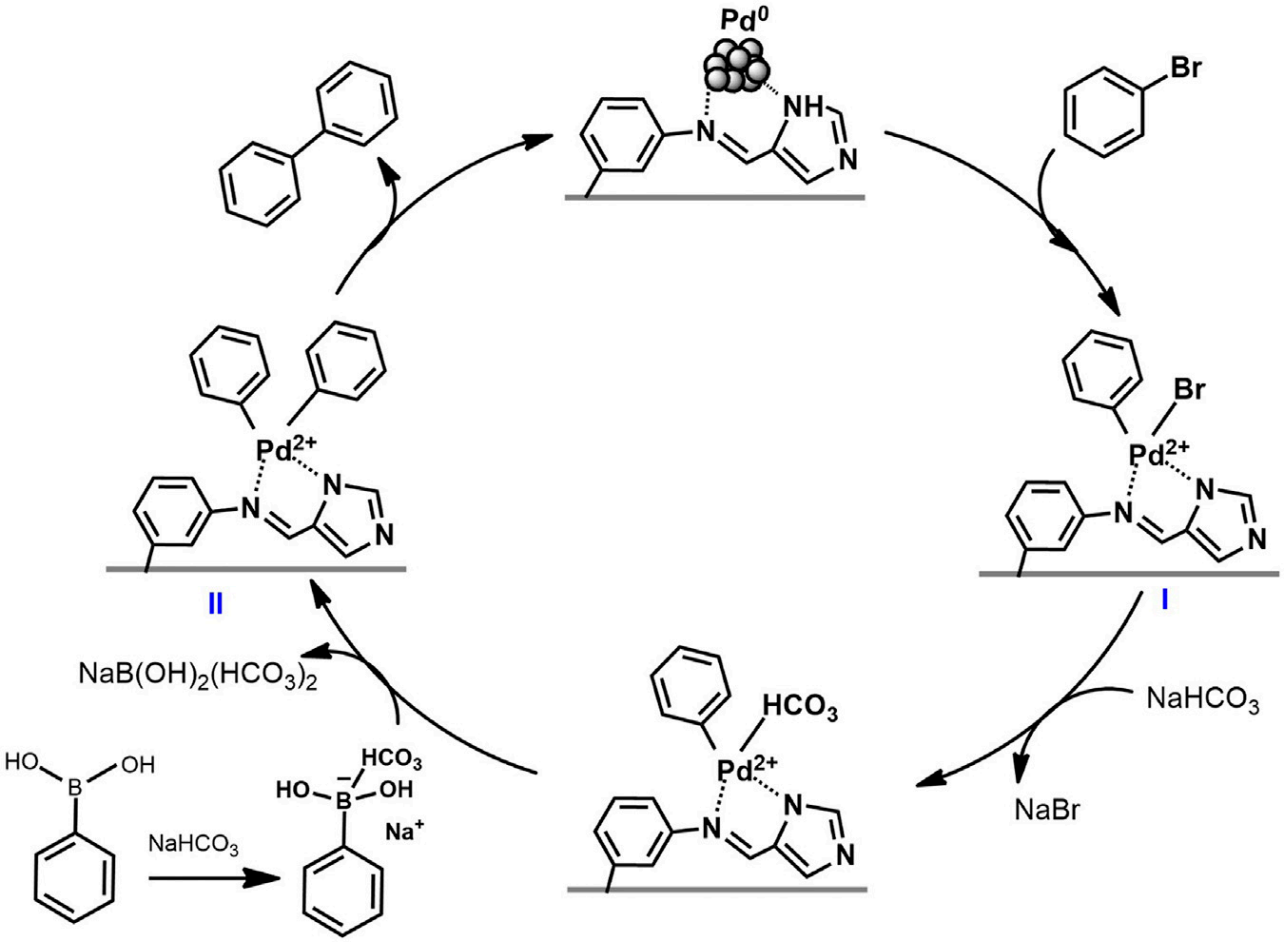
Catalytic cycle for the Pd^0^@UIO-66–SB–Im-catalyzed Suzuki coupling reaction.

A reaction route of the Suzuki coupling catalyzed by Pd^0^@UIO-66–SB–Im is proposed by combining the catalytic results and previous mechanism studies (Lin et al., 2012; Shang et al., 2014). As shown in Scheme 2, in the first step, Pd^0^ clusters stabilized by the Schiff base and imidazole groups on the surface of UIO-66–SB–Im interact with the electrophilic reagent bromobenzene to form organo-palladium species (I) by oxidative addition. Then the organo-palladium species (I) reacts with the other reactant phenylboronic acid through transmetalation in the presence of a base to produce an intermediate (II). Finally, biphenyl is produced, and Pd(II) is reduced to the original Pd^0^ state through the reductive elimination of (II).

## Conclusion

Highly dispersed Pd^0^ clusters anchored on MOFs was successfully synthesized by postsynthetic modification. The surface groups of Schiff base and imidazole groups that grafted on the MOFs are found to play a vital role in the control of small Pd clusters with an average size of only 1.5 nm. The catalyst Pd^0^@UIO-66–SB–Im exhibited excellent performance in the Suzuki coupling reaction of halogenated benzene with phenylboronic acid. Moreover, the catalyst behaved well with substrate compatibility and good reusability.

## Data Availability

All datasets generated for this study are included in the article/Supplementary Material.
