# Supplementary material for: Pd Clusters on Schiff Base–Imidazole-Functionalized MOFs for Highly Efficient Catalytic Suzuki Coupling Reactions
Source: Front Chem. 2022 Mar 1;10:845274. doi: 10.3389/fchem.2022.845274 (PMC8921604; doi:10.3389/fchem.2022.845274)
Supplement: Supplementary file 1 [file DataSheet1.docx]

Supplementary Material

Pd Clusters on Schiff Base-Imidazole-Functionalized MOFs for Highly Efficient Catalytic Suzuki Coupling Reactions

Yangqing Liu^1#^, Jingwen Sun^1#^, Lan Fan^2^, Qi Xu^1^*

^1^ School of Chemistry and Chemical Engineering, Key Laboratory under Construction for Volatile Organic Compounds Controlling of Jiangsu Province, Yancheng Institute of Technology, Yancheng, 224000, PR China

^2^Yancheng Lanfeng Environmental Engineering Technology Co., LTD, YanCheng, 224051, PR China

^#^These authors contributed equally to this work

*** Correspondence:**Qi Xu
ycxqsteve@163.com

**FIGURE S1｜**PXRD diffraction patter of the recovered Pd^0^@UIO-66-SB-Im.

**FIGURE S2｜**FTIR spectrum of the recovered Pd^0^@UIO-66-SB-Im.


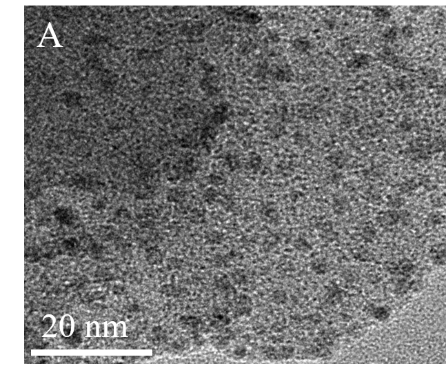


**FIGURE S3｜**(A) TEM image, and (B) Pd NPs size distribution of the recovered Pd^0^@UIO-66-SB-Im.
